# Protein folding as a driving force for dual protein targeting in eukaryotes

**DOI:** 10.3389/fmolb.2014.00023

**Published:** 2014-11-25

**Authors:** Bella Kalderon, Ophry Pines

**Affiliations:** ^1^Department of Microbiology and Molecular Genetics, Faculty of Medicine, Institute for Medical Research Israel-Canada, Hebrew University of JerusalemJerusalem, Israel; ^2^CREATE-NUS-HUJ Cellular and Molecular Mechanisms of Inflammation Program, National University of SingaporeSingapore, Singapore

**Keywords:** echoforms, membranes, organelles, signal peptide, MTS (mitochondrial targeting sequence), chaperones, reverse translocation, retrotranslocation

## Abstract

It is well documented that in eukaryotic cells molecules of one protein can be located in several subcellular locations, a phenomenon termed dual targeting, dual localization, or dual distribution. The differently localized identical or nearly identical proteins are termed “echoforms.” Our conventional definition of dual targeted proteins refers to situations in which one of the echoforms is translocated through/into a membrane. Thus, dual targeted proteins are recognized by at least one organelle's receptors and translocation machineries within the lipid bilayer. In this review we attempt to evaluate mechanisms and situations in which protein folding is the major determinant of dual targeting and of the relative distribution levels of echoforms in the subcellular compartments of the eukaryotic cell. We show that the decisive folding step can occur prior, during or after translocation through the bilayer of a biological membrane. This phenomenon involves folding catalysts in the cell such as chaperones, proteases and modification enzymes, and targeting processes such as signal recognition, translocation through membranes, trapping, retrotranslocation and reverse translocation.

## Introduction

**Figure 1:**
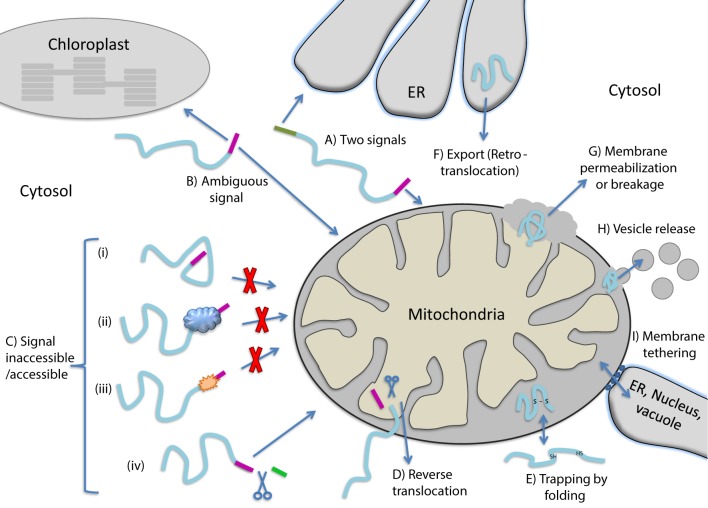
**Mechanisms allowing dual targeting of a single translation product**. **(A)** Competition between two signals for different organelles on the same polypeptide. **(B)** An ambiguous targeting signal is recognized by two organelles **(C)** Changes in the targeting signal accessibility caused by protein **(i)** folding, **(ii)** binding to cellular factors, **(iii)** modification or **(iv)** cleavage by a protease that exposes a targeting signal. **(D)** Reverse translocation, polypeptides move back to the cytosol during translocation into an organelle. **(E)** Trapping of proteins in an organelle by folding. **(F)** Export of proteins out of an organelle. **(G)** Release of proteins from organelles due to membrane permeablization or breakage. **(H)** Release of proteins from organelles via vesicles. **(I)** Release of proteins from organelles through tethering of membranes.

Dual localization of proteins can be achieved by a variety of molecular mechanisms all described in depth in reviews on this topic (Karniely and Pines, 2005; Regev-Rudzki and Pines, 2007; Avadhani, 2011; Yogev and Pines, 2011; Duchene and Giege, 2012; Carrie and Small, 2013; Carrie and Whelan, 2013). The dual localized, identical or nearly identical proteins, are termed “echoforms” indicating repetitious forms of the same protein distinctly placed in the cell (Yogev and Pines, 2011). Dual targeting mechanisms can be divided into two types, according to the number of translation products involved. Dual targeting by two translation products can occur due to the existence of multiple mRNAs that are derived from a single gene. This can be achieved either by alternative transcription initiation or mRNA splicing, in which the coding for a targeting sequence is removed. One mRNA can also give rise to several proteins by translation initiation from a downstream in frame start codon or stop codon read-through (see reviews above and Mitrpant et al., 2009; Freitag et al., 2012). In all these cases, two translation products (one containing and one lacking the targeting signal) are made and are targeted to different cellular locations. Thus, dual targeting is determined prior to synthesis of the protein(s) and these mechanisms do not necessitate a decision involving protein folding. Dual targeting of a single translation product on the other hand may or may not include protein folding as a driving force. In the next section we consider the participation of protein folding in the dual targeting mechanisms of single translation products. Figure 1 presents types of dual targeting mechanisms (Figures 1A–F) and other theoretically possible mechanisms (Figures 1G–I) that could lead to dual localization of echoforms in eukaryotic cells.

## Considerations of protein folding and dual targeting

**Figure 2:**
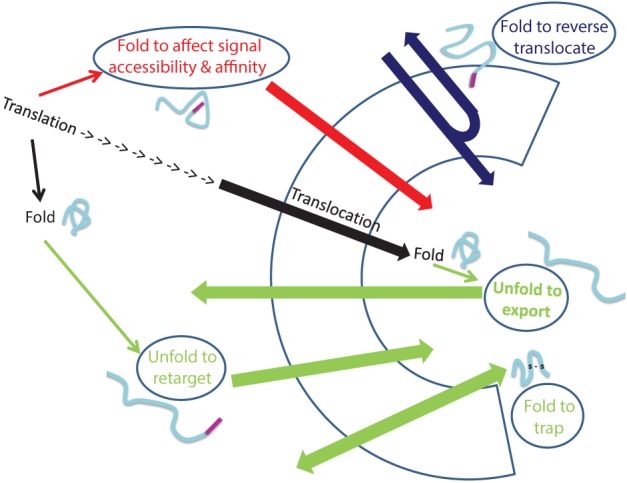
**Folding and unfolding “decisions” that determine dual targeting**. The normal process of protein translocation into an organelle (such as the ER and mitochondria) is depicted in black. Dual localization of proteins may be determined by folding of proteins, indicated by ellipses, prior to their targeting to an organelle (Red arrow), during translocation through membranes (Blue arrow) or after translocation into an organelle (Green arrows). Folding of proteins can affect signal accessibility and affinity (Red arrow; e.g., Adk1, Apn1, Gus1, CYP1A1, Fis1, b5R, CYP2E1), cause reverse translocation (Blue; e.g., Fum1, Aco1), trap proteins in an organelle (Green bottom arrow: e.g., Ccs1, COX19) or by unfolding, allow export of a protein out of an organelle (Green: top arrow, e.g., PrP^c^) or allow retargeting of a protein into an organelle (Green middle arrow, e.g., TERT, p53).

Dual localization of proteins may be affected by folding of proteins prior to their targeting to an organelle, during translocation through membranes or even after translocation into an organelle (Figure 2). In the first situation, dual targeting can be determined for instance by an ambiguous targeting sequence on a single polypeptide that can be recognized by more than one organelle (Figure 1B). Similarly, two (or more) targeting signals on a single polypeptide can provide a mechanism of dual targeting (Figure 1A). Here the balance of echoform amounts between the different organelles is determined by the affinity of each signal for its target. In these cases single translation products harboring one or more specific targeting signals can be dual targeted, when one of the signals is inaccessible under certain conditions or there is a change in the affinity of the signal for its receptor. We have chosen to present examples in which this change in accessibility or affinity is probably due to protein folding, or related processes which affect protein conformation such as modification or binding of another protein (Figure 1C).

An intriguing example in which dual targeting is determined by protein folding during translocation is the mechanism termed “reverse translocation” (Figures 1D, 2). In this case all protein molecules are first targeted to an organelle, begin their translocation but then a sub-population of the molecules moves back to the cytosol. Protein folding and assembly appears to be the driving force for this dual targeting and we will review this matter employing yeast fumarase as a paradigm.

We can identify a number of situations in which dual targeting is determined after translocation of substrates into organelles. For instance, newly synthesized proteins that fail to achieve their native active conformation in the endoplasmic reticulum (ER) are recognized and degraded by the ubiquitin–proteasome machinery which functions as a quality control system. This process is termed ER−associated degradation (ERAD) and is characterized by incorrect protein folding. Given that ubiquitin, ubiquitination machinery and proteasomes are not in the ER lumen and are associated with the cytosol and nucleus, ERAD substrates must be exported from the ER lumen and/or extracted from the ER membrane. We will discuss whether this can be considered as a basic mechanism for dual targeting of proteins (Figure 1F).

Dual targeting can also potentially occur after translocation of proteins such as the small TIMs, and superoxide dismutase (SOD). These proteins are imported into the mitochondrial intermembrane space, there they are trapped by folding and intramolecular disulfide bond formation (Figure 1E). In the absence of disulfide bond formation some of these protein molecules can move back through the TOM complex into the cytosol. In these cases, if such a protein is dual localized to the IMS and cytosol, one may refer to a mechanism of dual targeting involving “trapping of proteins” in a subcellular compartment due to protein folding.

How certain mitochondrial matrix proteins, under specific circumstances, can also be located outside the organelle has not been completely understood. This is troubling since in contrast to ERAD, above, a defined export system of proteins out of mitochondria or chloroplasts has not been detected. For example mitochondrial matrix proteins found outside mitochondria include the molecular chaperones mtHsp60 and mtHsp70 in mammalian cells and WHIRLY in plants (Soltys et al., 1996; Chandra et al., 2007; Kaul et al., 2007; Iosefson and Azem, 2010; Krause et al., 2012). Mechanisms that may explain the above observations include (i) the breakdown of the outer membrane or both inner and outer membranes, for instance during apoptosis (Figure 1G) (reviewed in Kluck et al., 1997; Green and Kroemer, 2004; Brenner and Mak, 2009), (ii) release of proteins by vesicles (Figure 1H, e.g., McLelland et al., 2014; Sugiura et al., 2014), or (iii) transfer between organelles by tethering of membranes such as those of the ER, vacuole/lysosome and mitochondria (Figure 1I, e.g., Kornmann et al., 2009; Elbaz and Schuldiner, 2011; Elbaz-Alon et al., 2014). In these cases we cannot assess whether protein folding is involved let alone whether it is the driving force for protein distribution. Nevertheless, we should keep in mind that, these mechanisms can theoretically lead to dual targeting of proteins in eukaryotes.

Finally, targeting or localization of proteins to particular compartments in the cell is not an unchangeable process. Actually the change in the destination of a protein is one of the mechanisms that should allow response to changes within the cell or in its environment. In this sense, relocalization can result in dual targeting of proteins (Figure 2). We shall discuss examples in which relocalization is apparently determined by folding of the retargeted protein.

When discussing examples in most cases mechanisms as presented in Figure 1 may overlap. For instance a protein may have two signals (1A) in which one of the signals becomes inaccessible (1C). In such cases, for the sake of clarity, we will refer only to the mechanism which is defined by folding.

### Accessibility of targeting signals

Single translation products can be dual targeted due accessibility or inaccessibility of a targeting signal. For some of the molecules of a specific protein the signal becomes inaccessible, thus inhibiting the contact with the specific receptor (Figures 1C, 2). Inaccessibility of the targeting sequence can be the consequence of (i) folding of the protein (ii) interaction of the protein with other proteins, or (iii) modification of the polypeptide chain. The yeast adenylate kinase 2 (Adk1) is distributed between the mitochondrial intermembrane space and the cytosol. Its rapid folding into a protease resistant structure hinders the accessibility of targeting signals (N terminal and internal sequences) to the mitochondrial receptor and some of these molecules are therefore retained in the cytosol (Bandlow et al., 1998; Strobel et al., 2002). In fact, x-ray crystallographic analysis (Egner et al., 1987), shows that both the N terminus and internal import relevant sequences are concealed in the folded protein structure of the native conformation, and the protein cannot be imported post translationally. In this case, competition between folding and targeting determines protein localization (Figures 1Ci, 2). A relevant study by Pfeiffer et al. (2013) shows that certain signal peptides promote efficient ER import when artificially fused to α-helical domains, but target unstructured polypeptides to mitochondria. Thus, targeting is affected not only by the targeting sequence but also by the structure of the nascent chain to which these sequences are attached.

The import efficiency may also affect accessibility of signals and substrates. ATFS-1, a key regulator of UPR^mt^ (mitochondrial unfolded protein response), has both a nuclear localization sequence (NLS) and an MTS (Mitochondrial targeting sequence) that is essential for UPR^mt^ repression. Normally, ATFS-1 is imported into mitochondria and degraded, however, upon stress, reduction in mitochondrial import efficiency causes a percentage of unprocessed ATFS-1 to accumulate in the cytosol and traffic to the nucleus (Nargund et al., 2012; Pellegrino et al., 2014) In this regard, the import efficiency could also be affected by modification of the translocation apparatus which may in turn affect signal accessibility (Schmidt et al., 2011; Harbauer et al., 2014).

The protein Apurinic/apyrimidinic endonuclease 1 (Apn1) (Vongsamphanh et al., 2001) is a case in which the targeting signal becomes inaccessible by binding of another protein (Figure 1Cii). Apn1 has two targeting signals: a nuclear localization sequence (NLS) and an MTS. Apn1 is bound by the protein Pir1 thereby concealing its NLS and allowing more pronounced mitochondrial targeting of the protein by the MTS. Apn1 (above) like Gus1 (below) belongs to a group of dual targeted proteins that are nucleic acid-transacting proteins (e.g., DNA damage response and RNA metabolism enzymes) that perform similar activities in mitochondria and outside the organelle. Glutamyl-tRNA synthetase (Gus1), in yeast, displays a similar mechanism to Apn1, by which it is localized to the mitochondria and cytosol. Import into mitochondria is driven by an MTS like sequence located following the first 190 residues. Binding of Arc1p to Gus1 inhibits mitochondrial import and causes the accumulation of Gus1 in the cytoplasm, while absence of Arc1 leads to exclusive mitochondrial localization. Arc1 levels are regulated by a metabolic change from fermentation to respiration, in which a reduction in Arc1 leads to an increase in mitochondrial Gus1 import and mitochondrial protein synthesis (Frechin et al., 2009). Apn1/Pir1 and Gus1/Arc1 are examples of signals whose accessibility is determined by the binding of another protein (Figure 1Cii).

For CYP1A1, the polypeptide modification makes one of the signals inaccessible, by cleaving off that signal (Figure 1Civ). In this sense proteases (and other protein modification enzymes) are reminiscent of molecular chaperones that bind the substrate protein, affect its conformation and ultimately its fate, which in this case is its final destination. CYP1A1 contains an ER signal peptide at its N-terminus followed by a cryptic MTS. The majority of the signal-peptide containing molecules are, as expected, translocated into the ER, yet a quarter of the polypeptides are cleaved by a cytosolic protease. These latter molecules escape ER membrane insertion and concomitantly expose an active cryptic mitochondrial targeting sequence (Addya et al., 1997; Avadhani et al., 2011). Thus, CYP1A1 dual distribution can be regulated by inducing the protease (e.g., with b-naphthoflavone).

### Changes in affinity of targeting signals

Human Fis1 is a tail-anchored membrane protein that regulates the membrane fission of both peroxisomes and mitochondria. The C-terminus of this protein is an ambiguous signal, which is affected by the binding of another protein (Figure 1Cii). The C-terminal 26 amino acids bind Pex19, a peroxisomal membrane protein import factor or alternatively function as a mitochondrial tail anchor (Delille and Schrader, 2008). Even though, both targeting events to mitochondria and peroxisomes are dependent on the same C-terminal sequence, they seem to be independent; down-regulation of Pex19 reduces peroxisome targeting but not targeting to mitochondria.

The affinity of an ambiguous targeting signal to the separate subcellular compartments can be modulated by protein modification (Figure 1Ciii). Modification of the mammalian NADH–cytochrome b(5)reductase (b5R) ambiguous targeting sequence, is a good example for this mechanism (Colombo et al., 2005). b5R is found both in the outer mitochondrial and ER membranes. Within the ER membrane, b5R is involved in lipid metabolism through its function as an electron acceptor. Within the mitochondrial outer membrane b5R mediates the regeneration of ascorbate from ascorbate free radical and is involved in transfer of electrons from cytosolic NADH to cytochrome C in the intermembrane space. b5R is translated as a single translation product from a single mRNA. This product contains an N-terminal targeting signal, required for targeting both to the ER and mitochondria. The targeting signal consists of a myristoylation consensus sequence followed by a 14 amino acids sequence which is moderately hydrophobic. The myristoylation consensus sequence is modified in about half of the b5R molecules. Nascent chains of b5R that are not myristoylated, remain bound to the signal recognition particle (SRP) and are consequently translocated into the ER. However, myristoylation of the N-terminal signal lowers its affinity for SRP, situating nascent chains on membrane free polysomes which are consequently imported into mitochondria (Colombo et al., 2005).

A change in the relative affinity of each targeting sequence for its target can also be affected by protein phosphorylation, which can change the relative amounts of the subcellular populations of the protein (Figure 1Ciii). A number of Cytochrome P450 monooxygenases (CYPs), mainly by the comprehensive studies of Avadahni and colleagues, have been shown to be dual targeted (e.g., see the CYP1A1 in the previous section). Phosphorylation plays a major role in the dual targeting of CYP2B1, CYP2E1, and CYP2D6 (Avadhani et al., 2011). CYP2E1, for example, which plays an important role in alcohol-induced toxicity and oxidative stress, is dual targeted to the ER and mitochondria. It contains an N-terminal 30 amino acids that constitute a bimodal signal for dual targeting. The model of dual targeting proposes that the low affinity of the CYP2E1 signal sequence for the SRP, causes half of the nascent chains to escape ER targeting and their translation as membrane free protein. Notably, CYP2E1 also harbors a cryptic signal located at the N-terminus of the protein (Robin et al., 2002; Avadhani et al., 2011). The twist in this case is that, cAMP-dependent phosphorylation of CYP2E1 on Ser129 by PKA results in the activation of the cryptic targeting signal, which increases the association of the protein with the cytoplasmic Hsp70 and Hsp90 chaperones and in turn binding to mitochondrial translocase subunits TOM70 and TOM40, thereby favoring its mitochondrial import (Anandatheerthavarada et al., 2009; Avadhani et al., 2011).

### Reverse translocation out of an organelle (during translocation)

An exceptional dual targeting mechanism is based on retrograde movement of a protein during its import (Figure 1D). The enzyme fumarase in the yeast *S. cerevisiae* is a paradigm of this mechanism. Fumarase is a TCA cycle enzyme in mitochondria and functions in the DNA damage response in the cytosol/nucleus (Yogev et al., 2010). In this case, *all* molecules are first targeted to mitochondria, begin their translocation and are processed by Mitochondrial Processing Peptidase (MPP). Nevertheless, a sub-population of the molecules moves back to the cytosol (Sass et al., 2001). According to the model, the driving force for this distribution is protein folding; if during import the nascent chain starts to fold in the mitochondrial matrix, it will complete its import and be localized in mitochondria. On the other hand, if the nascent chain starts its folding in the cytosol, thereby blocking its forward movement, the protein will withdraw from the import machinery, and will be localized in the cytosol (Sass et al., 2003). An indication of this mechanism are identical MPP processed echoforms found both inside and outside the organelle (Sass et al., 2001). The relative distribution of fumarase between the mitochondria and cytosol can be affected by the level of Hsp70 molecular chaperones in these respective compartments and by the rate of mitochondrial import (Karniely et al., 2006; Yogev et al., 2007; Regev-Rudzki et al., 2008). In addition, mutations or short deletions within the polypeptide sequence which disturb the fumarase structure (required for reverse translocation) cause full import of the protein (Sass et al., 2003). These data support the notion that folding is the major driving force for reverse translocation. Recently this model gained additional support; the bacterial fumarase homolog, fumC, was evolved by *in vitro* evolution into a dual targeted protein in yeast, suggesting that the natural folding of this protein was harnessed by evolution to distribute the protein in the cell (Burak et al., 2013). Worth pointing out is the fact, that upon expression of fumarase from the mitochondrial genome, no fumarase is detected outside the organelle, ruling out export or release of fumarase from mitochondria as the mechanism of dual targeting (Yogev et al., 2010). Dual targeting in *S*. *cerevisiae* of Nfs1 and Aco1 has been suggested to occur via the reverse translocation mechanism. Aconitase is targeted to the mitochondria and the cytosol, while Nfs is detected in the mitochondria and the nucleus (Regev-Rudzki et al., 2005; Naamati et al., 2009; Ben-Menachem et al., 2011a). Of importance is the finding that the MTS strength can determine the relative distribution of aconitase (and fumarase) between the two locations. The mechanism suggested is that the mitochondrial-targeting signal affects the translocation rate thereby determining the time (opportunity) required for the protein to fold or bind factors in the cytosol that block import (Regev-Rudzki et al., 2008). Consequently, slowing down translocation by reducing membrane potential or by using translocase mutant causes accumulation of cytosolic fumarase.

### Trapping of proteins in an organelle by protein folding (Figure 1E)

Numerous proteins of the intermembrane space (IMS) are imported by the mitochondrial disulfide relay system. These polypeptides which lack MTSs are recognized and oxidized by the IMS located receptor Mia40. Reoxidation of Mia40 is facilitated by the sulfhydryl oxidase Erv1 and the respiratory chain. The majority of the substrates of the mitochondrial disulfide relay system are small proteins with simple helix-loop-helix folds in which the helices are connected by two disulfide bonds; twin CX_3_C and twin CX_9_C proteins (Chacinska et al., 2009; Herrmann and Riemer, 2012). Other IMS proteins containing disulfide bonds include the yeast superoxide dismutase Sod1 and its copper chaperone Ccs1, both which are dually localized in the IMS of mitochondria and the cytosol. Sod1 and Ccs1 form part of the anti-oxidative system that dismutates superoxide anions to hydrogen peroxide. Sod1 is a dimeric copper- and zinc-containing protein that contains one disulfide bond per subunit. The insertion of this disulfide bond and of the copper ion is facilitated by Ccs1 (Sturtz et al., 2001; Reddehase et al., 2009; Kloppel et al., 2010, 2011; Gross et al., 2011) whose mitochondrial form also contains a stable disulfide bond between cysteine residues C27 and C64. In the absence of these cysteines, the levels of Ccs1 and Sod1 in mitochondria are strongly reduced. Accordingly, enhanced Ccs1 levels lead to an increase in the levels of active Sod1. Thus, the Mia40/Erv1 disulfide relay system introduces a structural disulfide bond in Ccs1 between the cysteine residues C27 and C64, thereby trapping Ccs1 in the IMS of mitochondria and controlling its distribution between the IMS and the cytosol. The distribution of Ccs1, in turn, determines the distribution of Sod1 by determining its oxidation and copper binding.

The cytochrome oxidase assembly factor COX19 which contains a twin Cx_9_C motif partitions between mitochondria and the cytosol in human cells (Nobrega et al., 2002; Leary et al., 2013). The cytosol is relatively enriched for COX19 when intracellular copper concentrations are elevated, suggesting that trapping of COX19 in the IMS is affected by copper levels. Proper function of SCO1 and SCO2 within the IMS is essential for the COX19-mediated transduction of appropriate redox signals outside mitochondria to regulate cellular copper homeostasis. The full mechanism of how copper affects disulfide formation and trapping of COX19 remains to be determined.

### Export of proteins out of organelles

Quality control of protein folding inside the ER includes chaperone-mediated assistance in folding and the selective targeting of terminally misfolded species to a pathway called ER-associated protein degradation, ERAD. Once selected for ERAD, substrates will be transported (back) into the cytosol, a step called retrotranslocation. Although still ill defined, retrotranslocation likely involves a protein conducting channel that is in part formed by specific membrane-embedded E3 ubiquitin ligases. A common mechanism of how individual misfolded proteins in the ER are first recognized is not known but it involves the exposure of hydrophobic domains on these proteins and the binding to chaperones. The selected substrates are then targeted to the membrane-embedded E3 ligase complexes where they undergo ubiquitination on their cytosolically exposed protein domains during or after retrotranslocation. Ubiquitin on substrates was originally thought to be a permanent modification that promotes the late steps of retrotranslocation. However, not all ERAD substrates are ubiquinated and degraded (Bernardi et al., 2010; Li et al., 2010). The enzymatic A1 chain of cholera toxin in the absence of ubiquitination retrotranslocates across the endoplasmic reticulum membrane into the cytosol, where it induces toxicity. The force that drives this retrotranslocation is not known. Viruses, like simian virus 40 (SV40), travel to the ER through the secretory pathway and then these viruses exploit ERAD components to reach the cytosol and nucleus (Tsai et al., 2001; Schelhaas et al., 2007; Bernardi et al., 2008).

PrP^C^ is a GPI-anchored secretory pathway protein which is found on the cell surface, in endocytic vesicles and endosomes. Several functions have been proposed for PrP^C^, including roles in cell adhesion, neurite outgrowth, neuronal excitability and neuroprotection. Whereas the proposed functions of PrP^C^ are a matter of controversy the fact that the protein can be detected in the endomembrane system and the cytosol is widely accepted (Biasini et al., 2012). The identification of interacting proteins with PrP^C^ which are cell surface or secreted molecules but also cytoplasmic, supports this conclusion (Biasini et al., 2012). Studies have shown that treating cells with proteasome inhibitors causes ubiquitylated PrP^C^ to accumulate, implying that PrP^C^ is degraded by the proteasome in the cytosol (Ma and Lindquist, 2001; Yedidia et al., 2001). In addition the PrP^C^ that was detected in the cytosol appeared to have undergone post-translational modifications in the ER (removal of N-terminal signal peptide and C-terminal GPI anchor signal sequence) strongly suggesting that PrP^C^ was reaching the cytoplasm via retrotranslocation. Like other ERAD substrates one can assume that it is the conformation or folding of the PrP^C^ molecules that determines retrotranslocation out of the ER (Figure 1F).

### Retargeting of echoforms

Dual or exclusive localization of a protein to specific compartments in the cell is a changeable process. In fact protein relocalization is one of the mechanisms that allows response to changes within the cell or in its environment. Recently, changes in the localization of hundreds of proteins have been shown to occur in response to different stress conditions in yeast (Breker et al., 2014). TERT, the enzyme telomerase reverse transcriptase is required to counteract shortening the ends of chromosomes in the cell nucleus. Nevertheless, there is evidence for a TERT function in mitochondria where it is proposed to reduce reactive oxygen species, protect mitochondrial DNA and reduce apoptosis. TERT harbors a bipartite NLS (nuclear localization sequence) which can be phosphorylated on a serine residue by protein kinase B/Akt (Chung et al., 2012). TERT also contains an NES (nuclear export signal) at its C-terminus, which can interact with the nuclear export receptor CRM1/exportin 1. Besides the NLS and NES, an N-terminal mitochondrial targeting sequence (MTS) has been identified on TERT (Santos et al., 2004). Mitochondrial TERT to a large extent is found in the matrix and IMS there it interacts with the mitochondrial translocases of the outer membrane Tom20 and Tom40 and Tim23 of the inner membrane and binds mitochondrial RNAs (Haendeler et al., 2009; Sharma et al., 2012). Upon oxidative stress, TERT is excluded from the nucleus and imported into mitochondria in which phosphorylation plays a regulatory role (Ale-Agha et al., 2014). Based on the decrease in nuclear TERT and concomitant mitochondrial targeting, the model is that the protein is exported from the nucleus and imported into the mitochondria (Figure 1Ciii).

A second possible example for protein relocalization is p53, which is a transcription factor that mediates apoptosis by transcription activation of pro-apoptotic genes or repression of anti-apoptotic genes. In response to stress, a fraction of p53 rapidly localizes to mitochondria prior to p53 nuclear accumulation, triggering mitochondrial outer membrane permeabilization and caspase activation (Mihara et al., 2003; Erster et al., 2004; Zhao et al., 2005). Whether mitochondrial p53 is derived from a cytosolic or nuclear pool of p53 molecules, remains to be determined. p53 can interact with mitochondrial proteins located in the mitochondrial matrix, such as the DNA polymerase γ, which is involved in the synthesis and repair of mtDNA (Bergeaud et al., 2013). In fact this interaction accounts for a higher mtDNA copy number in wild type vs. p53 knockout cells (de Souza-Pinto et al., 2004). Most of the intramitochondrial pool of p53 is present in two soluble compartments of mitochondria, the intermembrane space (IMS) and the matrix. Even though p53 is detected within mitochondria, the p53 polypeptide sequence lacks a mitochondrial-targeting signal, which is true for other nuclear factors that are detected in mitochondria such as CREB, NFκ B, or STAT3 (Cogswell et al., 2003; Lee et al., 2005; Wegrzyn et al., 2009). p53 can be targeted to the translocase of mitochondria by other proteins such as mtHsp70, Tid1, mitochondrial helicase RECQL4, or OKL38 (Marchenko et al., 2000; Ahn et al., 2010; De et al., 2012; Hu et al., 2012). p53 contains several reactive cysteines and may also be imported into mitochondria via the disulfide relay import system which was referred to in previous sections of this review (Herrmann and Riemer, 2012) Thus, although we do not know the precise molecular events by which p53 is retargeted to mitochondria this obviously involves protein binding to p53, unfolding and folding of p53 that are required for its import (Figure 2).

## Concluding remarks

It is now established that dual targeting of proteins in eukaryotic cells is a highly abundant phenomenon (Introduction and (Dinur-Mills et al., 2008; Ben-Menachem et al., 2011b; Yogev and Pines, 2011). Why are proteins dual localized and why is this phenomenon so abundant? We have discussed this question (Ben-Menachem et al., 2011b; Kisslov et al., 2014) and have concluded that dual targeting is driven by dual function. There are numerous examples of separate functions of dual localized proteins in the different compartments. Recently, dual targeted proteins were shown to be more evolutionarily conserved (by a number of criteria) which is consistent with a double selective pressure based on dual function (Kisslov et al., 2014). In addition, homologous proteins in different organisms can be dual targeted by different mechanisms (e.g., Yogev et al., 2011) indicating that what matters is the function in the different compartments and not the mechanism by which they are targeted. Current research indicates that dual localization is not a sloppy or leaky process but it is rather based on precise molecular mechanisms and serves distinct functional requirements. The regulation of dual targeting has profound influence on the way we comprehend the control of gene expression and function in eukaryotes.

In this review we have discussed how dual targeting can be affected and in some cases controlled by protein folding. We show that dual targeting can be affected via protein folding through the action of chaperones, proteases, kinases/phosphotases, oxidases, ubiquitin-ligases/ubiquitylases and other modification enzymes and binding proteins. The decisive folding step in each case can occur prior, during or after translocation through the bilayer of a biological membrane (Figure 2). The concept that we present is that the cell can regulate the distribution of many proteins, in concert, by modifying the level and activity of these folding catalysts and folding conditions. With all that said, it is quite clear that we do not have the full picture of protein dual targeting mechanisms in the cell and moreover we are only beginning to understand the impact of protein folding on this phenomenon. For example, a lot is yet to be learned regarding dual targeting involving membrane permeabilization/breakage, vesicle release or membrane tethering (Figures 1G–I).

### Conflict of interest statement

The authors declare that the research was conducted in the absence of any commercial or financial relationships that could be construed as a potential conflict of interest.
